# Gaze tracking of large-billed crows (*Corvus macrorhynchos*) in a motion capture system

**DOI:** 10.1242/jeb.246514

**Published:** 2024-03-22

**Authors:** Akihiro Itahara, Fumihiro Kano

**Affiliations:** ^1^ Wildlife Research Center, Kyoto University, Kyoto 6068203, Japan; ^2^ Centre for the Advanced Study of Collective Behavior, University of Konstanz, Konstanz 78464, Germany; ^3^ Max-Planck Institute of Animal Behavior, Radolfzell 78315, Germany

**Keywords:** Attention, Corvids, Crow, Head movement, Motion capture system, Visual field

## Abstract

Previous studies often inferred the focus of a bird's attention from its head movements because it provides important clues about their perception and cognition. However, it remains challenging to do so accurately, as the details of how they orient their visual field toward the visual targets remain largely unclear. We thus examined visual field configurations and the visual field use of large-billed crows (*Corvus macrorhynchos* Wagler 1827). We used an established ophthalmoscopic reflex technique to identify the visual field configuration, including the binocular width and optical axes, as well as the degree of eye movement. A newly established motion capture system was then used to track the head movements of freely moving crows to examine how they oriented their reconstructed visual fields toward attention-getting objects. When visual targets were moving, the crows frequently used their binocular visual fields, particularly around the projection of the beak-tip. When the visual targets stopped moving, crows frequently used non-binocular visual fields, particularly around the regions where their optical axes were found. On such occasions, the crows slightly preferred the right eye. Overall, the visual field use of crows is clearly predictable. Thus, while the untracked eye movements could introduce some level of uncertainty (typically within 15 deg), we demonstrated the feasibility of inferring a crow's attentional focus by 3D tracking of their heads. Our system represents a promising initial step towards establishing gaze tracking methods for studying corvid behavior and cognition.

## Teaser

**Highlighted Article:** A crow's attentional focus can be assessed by 3D tracking of their heads using a newly established motion capture system and behavioral experiments.

## INTRODUCTION

### What are birds looking at?

Many behavioral studies assume that a focal animal looks at something relevant to its behavioral decisions while gathering critical information for its survival, such as during predator vigilance, prey pursuit, mate choice, individual/species recognition, communication and social learning. However, inferring an animal's attentional focus by observing the animal's head and eye orientations may not be as straightforward as it seems, and this is particularly true for birds (as pointed out by Land, 1999). One reason for this is that, unlike humans and other primates, birds typically do not fixate on a single visual target for prolonged periods but often switch to different eyes and different visual field regions when attending to the same object (Butler et al., 2018; Dawkins, 2002; Kane and Zamani, 2014; Land, 1999). Another reason is that there is a large amount of diversity in birds' visual field and retinal configurations, such as the size of binocular overlap and the angles of foveal projections, most likely as an adaptation to the species' unique ecological niche (Fernández-Juricic, 2012; Kulemeyer et al., 2009; Martin, 2007). Thus, although it is a common practice to infer a bird's attentional focus from its head orientations (eye orientations are typically not tracked except when the bird can wear an eye-tracker, as shown in Yorzinski and Platt, 2014), detailed knowledge about its visual field and retinal configurations specific to each bird species is crucial for making accurate inferences.

Despite the challenges involved, some studies have been successful in inferring the focus of a bird's attention under specific conditions. For instance, studies have shown that starlings (*Sturnus vulgaris*) and peafowl (*Pavo cristatus*) orient their foveas, or area centralis (functionally and structurally similar to the fovea, except that it lacks the distinctive pit structure characteristic of the fovea), towards a predator image during vigilance (Butler and Fernández-Juricic, 2018; Yorzinski and Platt, 2014). Similarly, terns (*Gelochelidon nilotica*), hawks (*Accipiter gentilis*) and falcons (*Falco rusticolus*, *F. rusticolus*/*Falco cherrug hybrids* and *Falco peregrinus*) orient their foveas towards prey before initiating an attack (Kane et al., 2015; Kane and Zamani, 2014; Land, 1999). Therefore, if we know the species' visual field configuration, including the angles of foveal projections, and if there is a clearly defined visual target, observing a bird orienting one of its foveas (or any other sensitive spots in its retina) towards the visual target would likely indicate that the bird is looking at it.

However, even when there is a clearly defined visual target, there typically remains ambiguity regarding whether the bird is using its binocular (frontal) field or its non-binocular (lateral) fields. In such cases, additional contextual clues typically help differentiate between these possibilities. These clues include the properties of the object, such as the distance and motion of the object, as well as the behavior of the bird. Specifically, in terms of the object distance, birds predominantly utilize their binocular visual field when controlling their beaks to manipulate objects in close proximity. For instance, pigeons (*Columba livia*) and large-billed crows (*Corvus macrorhynchos*) employ their binocular vision when pecking at objects (Goodale, 1983; Matsui and Izawa, 2019) and New Caledonian crows (*Corvus moneduloides*) when using twig tools (Troscianko et al., 2012). Pigeons and chickens (*Gallus domesticus*) orient their foveas towards salient objects or conspecifics when those visual targets are presented at a distance (|Dawkins, 1995, 2002; Kano et al., 2022). Regarding object motion, pigeons visually track slowly moving objects with their binocular fields and fast-moving objects with their non-binocular field (Maldonado et al., 1988). In terms of bird behavior, pigeons utilize their upper binocular visual fields during perching flights (Green et al., 1992). Male zebra finches (*Taeniopygia guttata*) direct their binocular fields towards females during courtship displays (Bischof, 1988).

The laterality of eye use also serves as an additional clue for inferring birds' attentional focus. Birds typically exhibit a slight preference for their right eye when attending to detailed features of stimuli and a slight preference for their left eye when attending to more global features of stimuli such as relational spatial information (see Rogers, 2008 for a review; Tommasi and Vallortigara, 2001). For example, Australian magpies (*Gymnorhina tibicen*) display a preference for using their left eye to assess a model predator before withdrawing from it, whereas they prefer their right eye when approaching it (Koboroff et al., 2008). Eurasian jays (*Garrulus glandarius*), Eurasian jackdaws (*Corvus monedula*) and two species of tits (*Parus palustris* and *Parus caeruleus*) prefer to use their left eye when remembering spatial locations and their right eye when remembering object-specific cues in an associative memory task (Clayton and Krebs, 1994). Thus, in these species, the differential use of eyes seems to be generally influenced by both the type of object or task as well as the behavior of the birds, which is considered to be related to their hemispheric specialization (but see Coimbra et al., 2014; Hart et al., 2000 reporting retinal asymmetry of certain species).

In summary, to infer a bird's attentional focus, the essential information includes the precise orientation of the bird's head (ideally also its eyes), the species-specific visual configuration, as well as the location of a potential visual target. Additionally, considering contextual clues, such as the distance and motion of the visual target, as well as the behavior of the bird, can be informative.

### Gaze of corvids

This study focused on the feasibility of gaze tracking in corvids. Corvids are well known for their advanced cognitive abilities and complex social behaviors (Baciadonna et al., 2021; Boucherie et al., 2019; Emery and Clayton, 2004; Fitch et al., 2010; Hunt, 1996). Their visually guided behaviors offer valuable insights into their perception, attention, and cognitive processes. For instance, previous studies demonstrated that ravens (*Corvus corax*), like many species of birds, follow the gaze of other individuals (Schloegl et al., 2007). Ravens and several other bird species even follow other individuals' geometric line of gaze by going behind a visual barrier, suggesting that they take the visual perspective of others (Bugnyar et al., 2004). Ravens and scrub jays (*Aphelocoma californica*, *Aphelocoma coerulescens*) demonstrate sensitivity to the visual access of other individuals during food-caching events, adjusting their caching behaviors accordingly (Bugnyar et al., 2016; Dally et al., 2006; Emery and Clayton, 2001). However, despite previous studies relying on the birds' head orientations to infer their attentional focus, the details about how these birds orient their heads toward specific visual targets remain largely unclear. No systematic attempt has been made to examine their head and eye movements during object tracking in corvid species, aside from several studies focusing on the visually guided behaviors during object manipulations in crows (e.g. Matsui and Izawa, 2019; Troscianko et al., 2012). As a result, there is no technique available to track their gaze directions equivalent to primate eye-tracking (Hopper et al., 2021). This knowledge gap potentially impedes further advancements in the field of corvid behavior and cognition.

### Methods for tracking head movements of birds

There are several techniques available for tracking a bird's head direction. The most basic technique involves using fixed standard cameras to record the focal bird's head angles relative to a visual target in the captured images (Bischof, 1988; Butler and Fernández-Juricic, 2018; Land, 1999; Maldonado et al., 1988). More recent studies have employed head-mounted cameras or eye trackers to investigate birds' foveal use with the individuals that can tolerate the attachment of such devices (Kane and Zamani, 2014; Yorzinski et al., 2013). Recently, motion capture systems have been utilized to examine the 3D head movements of pigeons by attaching lightweight markers to their heads (Jiménez-Ortega et al., 2009; Theunissen et al., 2016; Theunissen and Troje, 2017).

Most relevant to this current study, Kano et al. (2022) used motion capture systems to track pigeons' head orientations while they were presented with attention-getting objects. When the object fell within a close distance (roughly within 50 cm), they oriented the lower-frontal area of their visual fields to the objects. When the objects were presented at a further distance, they oriented their foveas to the objects. Although eye orientation was not tracked in this previous study, most objects were observed within a few degrees of the foveal projections in the visual field, which is likely due to limited eye movement in this species, typically within 5 deg (Wohlschläger et al., 1993).

### The aims of this study

We examined the use of the visual field by large-billed crows, a corvid species often studied for their social and visual behaviors (Izawa and Watanabe, 2008; Matsui and Izawa, 2019; Miyazawa et al., 2020). To achieve this, we utilized a newly established motion capture system capable of tracking head orientations with an accuracy of less than 1 deg (Itahara and Kano, 2022). Our main aims were to determine which parts of the visual field regions they orient to when presented with an attention-getting visual target, and what contextual cues influence the differential use of their left and right eyes and distinct visual field regions. By addressing these questions, our larger goal was to establish a gaze-tracking technique in a bird species. This advancement enables us to conduct gaze-tracking studies that have been conducted mainly in human infants and primates (Hopper et al., 2021), and should prove valuable for future studies on the behavior and cognition of birds.

Study 1 examined the visual field configuration of restrained crows using an established ophthalmoscopic reflex technique. Specifically, we assessed the width of the binocular fields and the extent of eye movement in this species. Rahman et al. (2006) examined ganglion cells, which receive visual information from photoreceptors and transmit it to the brain, in the retina of large-billed crows and determined that the highest densities of these cells are located in the center of the retina (area centralis), suggesting their projection (i.e. visual axis) is close to the optical axis, which is a line passing through the center of the cornea and the pupil, in this species. We thus also examined the angle of the optical axis in study 1.

In study 2, we tracked the head orientations of freely moving crows using a motion capture system. We then mapped the visual targets onto the reconstructed visual fields of the crows. Previous studies have shown that this species of crows relies on their binocular fields during and just before pecking (Matsui and Izawa, 2019). However, it remained uncertain how this crow species differentially uses their binocular and non-binocular fields when presented with objects at a distance beyond pecking range. Studies in pigeons have suggested that they utilize either their binocular or non-binocular fields based on the speed of moving objects (Maldonado et al., 1988). Hence, study 2 focused on examining how crows use both binocular and non-binocular (lateral) fields when they were presented with objects at a distance beyond pecking range. To examine differences based on object movement, we compared how the crows utilized their visual fields when the object was either in motion or stationary. When using their non-binocular field, it was expected that crows would direct their optical axis, which is close to their visual axis, towards visual targets. Additionally, we examined the laterality of eye use in crows. We predicted that our crows might show a right-eye preference because we presented them with small objects of various shapes and colors, which could motivate them to attend to the detailed features of the stimuli.

## MATERIALS AND METHODS

### Visual field configuration (study 1)

#### Subjects

Four large-billed crows were used in study 1 (see Table S1 for the details of the subjects). They were housed socially in a group of 12 crows in an outdoor aviary (130 m^3^) at Uki, Kumamoto, Japan. Nine were captured at a local crow trap (at Gyokuto, Kumamoto, Japan, from January to February 2021) and transferred to our facility courtesy of the farmer, three of which were captured at the study station using a standard crow trap (in February 2021, with permission from the local government, Kumamoto Prefecture; permission no. 02002). All birds were free-flying yearlings at the time of capture. The ages of the birds were estimated at the time of capture based on the pink coloration of their oral cavities, following a previous study (Miyazawa et al., 2020). Study 1 was conducted between September and October 2021. The crows were estimated to be 1–2 years old at the time of the study. The sex of the birds was determined by plucking 2–3 breast feathers and subjecting these samples to standard PCR tests (Fukui et al., 2008). They were provided opportunities to interact with conspecifics and various enrichment objects (dog toys, stones and branches). They had *ad libitum* access to water, except during the experiments (approximately 1 h on the day of the experiments) and were fed once daily with nutritionally rich foods (dog/cat food, meat, boiled eggs and wild plants). Three of the four crows (BO_21, BY_21 and YY_21) were used in study 2.

#### Ethical note

To capture wild crows, we used one section of our outdoor aviary (64 m^3^) as a crow trap while temporarily opening a one-way entrance (openings with hung wires) on top of the aviary. The local farmer used a standard (commercially available) crow trap (20 m^3^). The crows had *ad libitum* access to water and fresh meat in the traps. The traps were routinely checked by an experimenter or a local farmer. The crows were kept in the traps for a maximum of 1 day. None of the crows were severely injured during capture. A few crows had scratches, presumably from the wires at the one-way entrance. We provided veterinary care to the injured crows on such occasions. The crows captured by a local farmer were transported from the capture site to our aviary by car and kept in a dog carrier (or box of the same size) for approximately 1 h. The main experimenter (A.I.) was trained in March 2019 (more than 2 years before the onset of experiments) by another experimenter (F.K.) and a veterinarian to safely catch crows using a net in the outdoor aviary, handle them and check their condition. We caught crows for routine health checks daily and checked for any signs of distress after they were released back into the aviary, confirming that the catch itself did not seem to incur lasting stress in the crows. The time required to catch the crow was approximately 2 min.

The details of the experimental procedure are described below. We minimized the restraint time for visual field observations (maximally of 1 h daily) and the amount of breast feather sampling (plucking a few breast feathers for sex determination). After the experiment, they were released into the flock for the remainder of the day, and we confirmed that these procedures did not incur lasting signs of distress in the crows. The animals were maintained socially and had free access to food and water except during the experiments. When we found a sick crow, we provided this individual with veterinary care and subsequently isolated it from the flock (but within the visual and auditory access of the flock) in a calm place to rest. The study protocol was approved by the Institutional Committee of the Wildlife Research Center, Kyoto University (WRC-2021-009A).

#### Apparatus

**Fig. 1.**
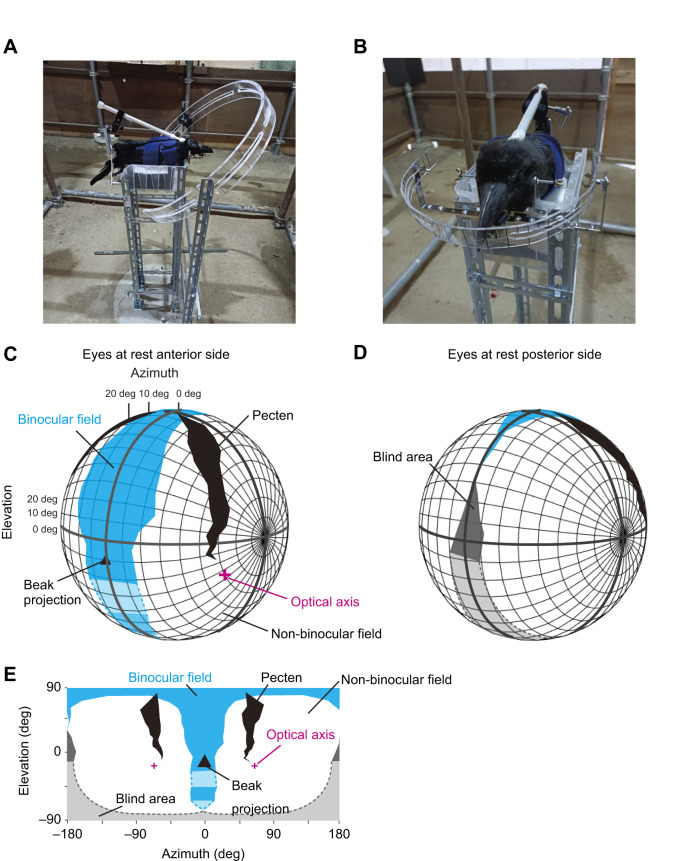
**Visual field configurations of large-billed crows (*Corvus macrorhynchos*).** (A) To observe retinal boundaries, a visual perimeter arm with a diameter of 560 mm was used. (B) To observe the optical axes a visual perimeter arm with a diameter of 180 mm was used. (C,D) 3D illustration of the visual field of large-billed crows in the head-centric polar coordinate system (at 10 deg intervals) with the eyes at rest (no attempt to induce eye movement). The boundaries of each visual field were calculated as the mean of the four participants. The illustrations show the binocular field (cyan), monocular field (white), blind area (gray), pectens (black), and positions of the optical axes (magenta) viewed from the subject's anterior (C) and posterior sides (D). The projection of the beak-tip (eye–beak-tip line) is denoted by a filled triangle. Dotted lines and lighter colors denote manually filled gaps in the measurements (when the visual field could not be observed because of obstruction by the equipment or the crow's body and wing) based on the shape of the head of the crow. (E) The same data are shown unfolded in 2D.

We used an established ophthalmoscopic reflex technique (Martin, 1986; Martin and Katzir, 1994). The apparatus comprised a cradle (plastic box stuffed with rubber foam and sponges) and a visual perimeter arm (bent transparent plastic plates; a diameter of 560 mm for observation of the visual field and a diameter of 180 mm for the observation of the optical axes; Fig. 1A,B). The cradle restrained the subject's body and head by wrapping the subject's entire body and both legs with soft fabrics/straps and fixing its head with a beak holder (made of metal frames, rubber sheet and thin wire), padded ear bars and padded rear-head bar (made of metal bars and silicon). These apparatus was covered/filled with sponges or silicone to prevent any harm to the crows. We ensured that their breathing remained undisturbed while they were restrained. The perimeter arm was a semicircle that extended along the azimuth axis (left and right sides of the bird) and moved along the elevation axis (top and bottom sides of the bird). We positioned the midpoint of the subjects' eyes at the center of the perimeter arms (the origin of the head-centric polar coordinate system). We set the subject's lower mandible horizontally in the beak holder (and calibrated the subject's head in the data analysis such that the line connecting the midpoint of the two eyes and the beak-tip (eye–beak-tip line) matched the crows' natural perching postures observed in study 2; see below). The experimenter observed the reflection of the subject's retina using an ophthalmoscope (Welch Allyn Ophthalmoscope, Welch Allyn, NY, USA) at each angle on the perimeter arm. The crows were restrained for approximately 1 h.

#### Procedure

Study 1 was performed over 2 days for each subject. On the first day, the retinal reflections of the crows were measured while holding the ophthalmoscope on the visual perimeter arm (diameter 560 mm) when no eye movement was experimentally induced. To estimate the boundaries of the retina and pecten in each eye, we searched for the edge of the retinal reflection (the angle at which the ophthalmoscope image appeared half-bright and half-dark) in each eye at a step of 1 deg in azimuth (while smoothly moving the ophthalmoscope along the perimeter arm) and 10 deg in elevation (while moving the perimeter arm at a step of 10 deg in elevation). Owing to eye movement, we observed the edge of the retinal reflection in several azimuth angles (corresponding to the maximal amplitude of eye movement when the experimenter did not induce eye movement; see Results); we used the maximum angle of azimuth at each elevation as a representative score for each subject, consistent with Troscianko et al. (2012). These procedures were repeated until we completed the measurements of all elevation and azimuth angles, although we skipped a given observation angle when the bird's body/wings and the apparatus obstructed the view of the ophthalmoscope.

On the second day, we repeated the same procedure while experimentally inducing the subject's eye movement to measure the degree to which the induction of eye movement changed the maximum width of the binocular overlap (or blind area) and to estimate the maximum amplitude of the eye movement from the observed changes. While most previous studies induced bird's eye movement to estimate the range of the bird's visual fields when their eyes were either converged (eyes moving to the beak-tip) or diverged (eyes moving to the posterior of the head) (Baumhardt et al., 2014; Martin, 2007), we instead measured the maximum and minimum ranges of the visual field in any eye direction because of our interest in the 3D eye movements of freely moving crows in study 2. Thus, we induced eye movement either toward or away from the view of the ophthalmoscope along the perimeter arm (the azimuthal axis; Fig. S1). To induce eye movement in crows, while most previous studies (cited above) used light spots or tapping sounds, we presented a small object (a fabric ball with a diameter of 5 cm) while slightly wiggling it, as we found that this presentation method induced longer eye fixation and made observation easier. The measurement interval was changed from 10 deg to 30 deg for time efficiency (to maintain a total restraint time of approximately 1 h). Although this compromise in the resolution of measurement along the elevation axis may lower the resolution of the estimated overall shapes of binocular overlap (or blind area) (Potier et al., 2018), we did not change the measurement step of 1 deg along the azimuth axis. Thus, the interval of 30 deg along the elevation axis should be sufficiently large to answer our main questions here, namely, the changes in the maximum width of binocular overlap (or blind area) and the maximum amplitude of eye movement estimated from such changes.

After completing all measurements of the subject's visual field on the second day, we also measured the angle of the optical axes when no eye movement was experimentally induced. To achieve this, the original visual perimeter was replaced with a smaller perimeter (diameter=180 mm) to make it easier to observe the corneal and lens reflections. We searched for the specific angle at which the reflections from the anterior and posterior surfaces of the cornea and lens (the Purkinje images) were aligned using an ophthalmoscope (two of the four Purkinje images were prominently observed). We performed this at steps of 1 deg in azimuth and 10 deg in elevation and repeated this procedure at least nine times per eye for each subject. We used the median value of these repeated measurements as the representative score for each participant. All measurements were performed by the same experimenter (A.I.).

#### Data analysis

A standard visual field reconstruction procedure assumes a hypothetical, infinite viewpoint (Martin, 1984). However, as the ophthalmoscope is placed at a relatively close distance (280 mm, the radius of the 560 mm perimeter arm in our study), a viewpoint correction is necessary based on the distance between the nodal points of the two eyes. This distance was calculated using the distance between the surfaces of the two eyes and the relative location of the nodal point in each eye. The former was calculated for each individual using our custom structure-from-motion application (see the ‘Head calibration’ section of study 2 for details). Although the latter was not available for this species, a consistent value (2/3 of the distance between the surfaces of the two eyes) was used in a previous study across corvid species (Troscianko et al., 2012; confirmed also from personal communication); thus, we used the same value in this study.

Each subject's head was calibrated by rotating it in the data by aligning the line connecting the midpoint of the two eyes and the beak-tip (eye-beak tip line) to 0 deg in azimuth and −10 deg in elevation (using the 3D coordinates of the eyes and beak-tip calculated from our structure-from-motion application; Fig. 1C). A value of −10 deg was chosen because the vector connecting the midpoint of the two eyes and the beak-tip points to approximately −10 deg in the natural perching postures in study 2 (Fig. S2A). The subject's binocular field was calculated as the overlap of the left and right visual fields, and the blind area was calculated as the absence of either field. At some measurement points, the edge of the retinal reflection could not be observed owing to obstructions by the equipment or the body and wings of the crow. For visualization purposes in study 1 and reconstruction of the visual field model in study 2, we manually filled in the gaps in the measurements based on the shape of the head of the crow. This means that although the subject's body obstructed the visual fields in our measurement, we did not consider these obstructions as blind spots, considering that the body and wing positions can vary in unrestrained crows. Therefore, these manually corrected values were not used as the main results of study 1.

### Visual field use (study 2)

#### Subjects

A total of 11 large-billed crows were used in study 2 (Table S1). Study 2 consisted of three experiments. Experiment 1 tested four subjects in June–July 2020; Experiment 2 tested four subjects in May–June 2021; and Experiment 3 tested four subjects in August 2021 (one subject was used in both experiments 2 and 3). An additional six crows were not tested because they habitually removed the markers attached to their heads (see below for details). In experiment 1, crows were captured at the study station using a standard crow trap (in November 2018, with permission from Kumamoto Prefecture; permission no. 2). Crows in experiments 2 and 3 were captured by a local farmer or at the study station using a standard crow trap in 2021, as described above. Four of the 11 subjects were housed in an outdoor aviary (64 m^3^, expanded to 130 m^3^ in 2020) socially with a group of five in 2020, and the remaining subjects were housed similarly as described in study 1. They were estimated to be 1–3 years old during the study period.

#### Ethical note

Trapping and housing procedures were identical to those used in study 1. The details of the marker attachment are described below. Most of the crows immediately tolerated the attachment of lightweight markers. However, we excluded the crows that removed the markers. The motion capture room was connected to the aviary, and the door was left open before the experiment, allowing the crows to gradually acclimate to the room. To assess the acceptance of marker attachment and acclimate them to the procedure, we released each crow into the motion capture room several times before the experiments. The study protocol was approved by the Institutional Committee of the Wildlife Research Center, Kyoto University (WRC-KS-2020-12A and WRC-2021-012A).

#### Apparatus

**Fig. 2.**
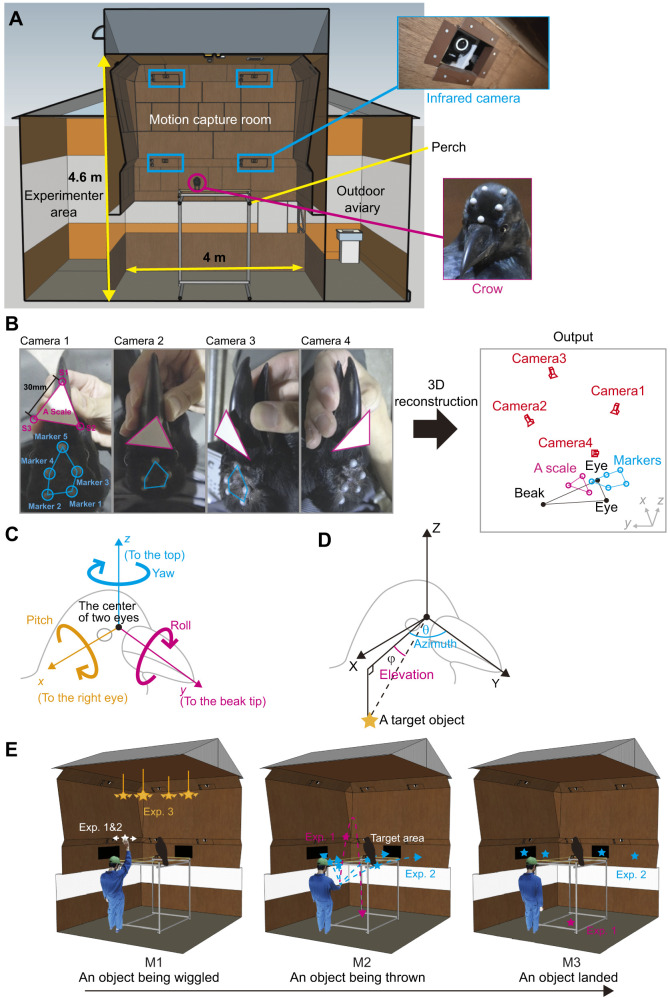
**Facilities and procedures used in this study.** (A) An illustration of the motion capture room (W: 4.0 m, D: 4.0 m, H: 4.6 m). The insets show the infrared camera and the crow's head with infrared reflective markers. (B) Our structure-from-motion application reconstructs 3D coordinates of key points from 2D images taken from multiple angles. (C) The reconstructed head-centric coordinate system. Thin arrows indicate *x*, *y* and *z* axes. Thick arrows indicate the direction of rotation in yaw, roll and pitch in this system. (D) The 3D position of a visual target is described in a polar coordinate system (azimuth and elevation). (E) Illustrations of three experiments and distinct movements of visual targets in study 2. Visual targets were wiggled (M1), thrown to (M2) and still (M3) on the ground (experiment 1) and the wall (experiment 2). Visual targets suspended from the ceiling were only wiggled (M1) (experiment 3).

Crows' head movements were tracked in a motion capture room built inside the aviary (called the ‘Corvid Tracking Studio’; W: 4 m, D: 4 m, H: 4.6 m, 73.6 m^3^). This system is optimized to track the fine-scale movements of crows, including head movements, while freely moving and interacting with objects or conspecifics. Unlike other labs that have built a motion capture system applicable to the tracking of various animal species (Nagy et al., 2023), we specialized our system for the tracking of crows because crows typically need long habituation periods in a new room (the room was therefore built inside the aviary) and also because the structure of the room was designed to prevent crows from perching in non-trackable areas (wall angle >90 deg) and from damaging the infrared cameras (the cameras were embedded inside the walls; Fig. 2A) (Itahara and Kano, 2022). A perch (W: 1.4 m, D: 1.8 m, H: 2 m) was installed at the center of the motion capture room such that crows could rest and interact with objects/conspecifics well above ground level. Twelve infrared cameras (Flex13, Optitrack, Corvallis, OR, USA; 1280×1024 pixels) were installed in the motion capture room; six cameras at a height of 2.5 m were aimed at the ground, and six cameras at a height of 4.5 m were aimed at the perch. The motion capture system recorded the 3D coordinates of the infrared reflective markers at 120 Hz (although our data were smoothed to 36 Hz during processing; see below). Three to five 6.4 mm (in diameter) markers with 3 mol l^−1^ 7610 reflective tape (6.4 mm M3 marker, Optitrack; approximately 0.1 g per marker) were attached to the subject's head with double-sided tape and glue, which weighed in total less than 1% of the crow's weight (680–900 g). The double-sided tape was affixed to the marker, and the other side of the tape was adhered to head feathers of the crows using glue. Motion capture software (Motive, Optitrack) controlled the cameras and recordings. Before each experimental session, the motion capture system was calibrated with a calibration wand (CW-500, Optitrack) and a calibration square (CS-200, Optitrack). A web camera (BRIO, Logicool, Japan; 3840×2160 pixels) was attached to the center of the ceiling to monitor the room and the general behavior of the crows.

#### Stimuli and procedure

Before each experimental session (one session per day per subject), an experimenter caught a subject crow with a net in the outdoor aviary, loosely restrained its legs with soft fabrics/strings, placed it on the experimenter's lap, and attached five 6.4 mm markers to the subject's head with double-sided tape and glue (the marker arrangement was as shown in Fig. 2A) and calibrated the subject's head (see below for the detailed procedure). The subject was then released into the motion capture system for approximately 30 min. Only crows that did not attempt to remove the markers participated in the experiments (see the Subject section). Because one crow (GG_20) repeatedly attempted to remove markers when five markers were attached to the head, we reduced the number of attached markers from five to three (the minimum number required to reconstruct 3D movement) for this crow.

In each session, after releasing a subject crow into the motion capture room, we first acclimated the subject for approximately 2 min until the subject settled on the perch. After acclimation, in experiments 1 and 2, the experimenter entered the motion capture room and stood stationarily at a predetermined location for 2 min before presenting the visual targets. As crows are generally sensitive to a human's direct gaze, the experimenter wore a baseball cap (with reflective markers to track the experimenter's position) to hide the experimenter's eyes from the crows. The experimenter wore a wristband with reflective markers on the right hand to track the experimenter's hand while holding the object. The experimenter then presented various attention-getting visual targets in the subject crow. In all experiments, the stimuli were small toys of different shapes and sizes (approximately 2–8 cm in diameter), such as artificial flowers, pocket tissue bags, felt cloths, plastic cups and kitchen sponges. A 9.5 mm reflective marker was attached to each object to track its position using the motion capture system during the experiments. To examine the potential effect of presentation height of the stimuli, we conducted three experiments, each varying in this regard (Fig. 2E).

In experiment 1, the experimenter took an object from a waist bag (after holding the hand inside the bag for 10 s), held it above the experimenter's head, wiggled it for 5 s, and threw it upward; the object followed a parabolic motion and then landed on the ground (approximately at the center of the room) below the eye level of the perched crow. Experiment 2 was identical to experiment 1, except that the experimenter threw an object onto one of the four target areas on the wall, approximately at the eye level of the perched crow. We attached Velcro tape to the wall and the objects such that the objects could land on the wall (see Movie 1). In experiment 3, four visual targets were suspended from the ceiling (approximately 3 m above ground level) and connected to the experimenter area via transparent fishing lines. The experimenter did not enter the room in experiment 3 but instead controlled the threads from outside the room (immediately after the 2 min acclimation of the subject crow). The experimenter wiggled each object individually for 5 s at intervals of approximately 2 min. In all experiments, each session repeated the presentation of a single object (a trial) ten times. Each day, a single session was conducted per participant. In Experiment 1, we performed 20 sessions per bird. Because the results from Experiment 1 indicated a relatively small variation in the crows' visual responses across sessions, we restricted the number of sessions and performed six sessions each in experiments 2 and 3. The order of testing for each crow was counterbalanced across the days. Each session lasted approximately 30 min. After each session, we removed the head markers from the subject's head by detaching the marker from the double-sided tape (to allow the subject to rest better outside the testing time) and released the subject into the aviary with its social group.

#### Head calibration

The head of the subject crow was calibrated using our custom structure-from-motion application, which reconstructed the positions of the morphological key points (eyes and beak-tips) from the head marker positions. Specifically, before each experimental session, the experimenter loosely restrained the bird (see ‘Stimuli and procedure') and successively filmed images of the head from four distinct angles using a standard RGB camera (FDR-AX40, SONY, 3840×2160 pixels) (Fig. 2B) (Itahara and Kano, 2022). To calibrate this camera (set at a manual focus mode to maintain consistent camera intrinsic parameters), we filmed a printed checkerboard image (64×44.8 mm, with each square sized at 6.4×6.4 mm, approximately the size of the crow's head) from 10–15 distinct angles. During calibration, a triangular scale (30 mm on each side) was temporarily attached to the root of the beak to provide information on the absolute distances between the marked points. The entire calibration process lasted for approximately 30 s.

The RGB camera was calibrated using the filmed checkerboard images in the ‘Camera Calibrator’ app (available in the Image Processing Toolbox) of MATLAB (MathWorks, Natick, USA); this process yielded the camera intrinsic parameters. Using these parameters, the row images were calibrated. In our custom application (GitHub: https://github.com/itaharaakihiro/head_orientation_calibration_app_set), we manually identified the 2D coordinates of the center of the infrared markers, and other key points (the center of the two eyes, the beak-tip, and the three corners of a triangular scale) in each image and then reconstructed the 3D coordinates of these points using the structure-from-motion algorithm.

Finally, from the obtained 3D coordinates of the eye centers and beak-tip, we defined the head-centric coordinate system (comparable to that used in study 1) with its origin located at the midpoint of the two eyes, the *x*-axis pointing to the center of the right eye, the *y*-axis pointing to the beak-tip, and the *z*-axis orthogonal to the *x*- and *y*-axes and pointing to the top of the head. Rotational head movements in yaw, roll, and pitch were defined as the rotation around the *z-*, *y-* and *x*-axes, respectively (Fig. 2C). We then rotated the head-centric coordinate system by −10 deg in pitch so that the *y*-axis was oriented to the horizon instead of the beak-tip, as the crows' eye-beak-tip line (the line connecting the midpoint of the two eyes and the beak-tip) pointed down at approximately −10 deg in their natural posture (Fig. S2A). We referenced the horizon rather than the beak-tip to reveal the crows' visual field use with reference to the environment (rather than their morphology). We calculated the angles (azimuth and elevation) at which the visual target was located in the head-centric coordinate system (Fig. 2D).

#### Motion capture data processing

In the Motive software, we first defined the rigid bodies (unique arrangements of markers consistent across frames) for the head of the crow and the experimenter's cap and wristband in a given frame. We then used this information to automatically label all the markers in all frames in the software. Because the software occasionally mislabeled the markers within each rigid body in certain frames (which caused an erroneous orientation of the rigid body), we manually corrected them. The data files for sessions 11 and 16 (out of 20 sessions) for participant BB_20 in experiment 1 malfunctioned and were therefore excluded from the analysis (all 20 sessions were used for the other three participants in experiment 1). Data filtering (noise removal, gap fill, and smoothing) was performed using motion capture software and our custom code (MATLAB), as detailed in Fig. S3. We determined the individual parameters by visually checking the time series of the data to minimize any impossible movement (biologically and physically) and missing values. The customized MATLAB code and sample data are available in our online repository (https://osf.io/3dmg7/?view_only=c0f0a272ebff466ca8d0a3792da3ab4e).

#### Saccade filter

A saccade was detected in frames where the axial angle speed exceeded 200 deg s^−1^ (Fig. S2B). This axial angle was calculated from the rotation matrix that converted the head angle recorded in a given timeframe into that recorded in the next frame. Although there was a population of small saccades (shorter than 50 ms and smaller than 5 deg) in our data, we excluded them from our analyses (and thus included them in the inter saccadic intervals) because they were not distinguishable from the motion capture noise in our data. We then filtered out the moments at which birds made head saccades (8.0% of all data) because visual processing is likely inhibited during saccades in birds (Brooks and Holden, 1973). Fig. S2C also shows histograms of saccade amplitude, inter-saccadic intervals and saccade durations.

#### Data extraction

**Fig. 3.**
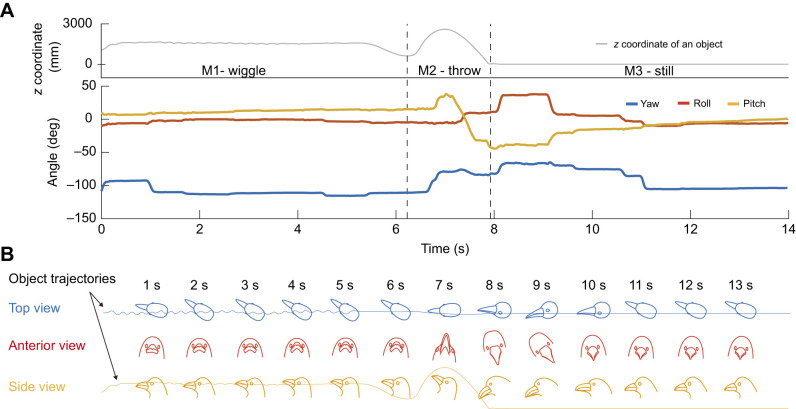
**An example of a crow's head movement when presented with a visual target thrown to the ground.** (A) The height of an object (*z* coordinate) and the rotational movements of a crow's head (in yaw, roll and pitch) are shown over a period of 14 s in experiment 1, where an object was wiggled (M1) thrown in a parabolic arc (M2) and then lay still on the ground (M3). (B) Illustration of the crow's head position using the same data.

To examine stimulus-driven attention in crows, we extracted the moment crows most likely attended to an object. Specifically, we extracted three critical time windows for object presentation; 1 s (120 frames) following the moment when the experimenter took out the object from the waist bag (defined as movement M1), 1 s following the moment when the experimenter threw the object (M2), and one second after the moment when the object landed on the ground/wall in experiments 1 and 2 (defined as M3), and 1 s following the moment when the experimenter wiggled the object suspended from the ceiling in experiment 3 (defined as M1) (Fig. 2E). The 1 s duration was chosen as it appeared to ensure sufficient response time following the onset of the stimuli and occurred well before the disengagement from the presented stimuli (see Fig. 3). At each session, the object was presented 10 times (trials), and we pooled all the data in each session per subject (10 s of data per session). We used a session (day) rather than a single-object presentation (trial) for a subject's response in the statistical analysis (see below) because the trials yielded too many zeros in our data (which would have unnecessarily complicated our statistical analysis).

#### Visual field definition

We used visual field configurations with the eyes at rest (with no attempt to induce eye movement) in study 1 (Fig. 1E) to examine the visual field use of the crows in study 2. We did not consider the projections of the pecten in the analysis of study 2 as blind spots because their projections could easily be moved by eye movements. To quantify binocular field use, we derived a binocular use score, defined as (binocular looking time–non-binocular anterior looking time)/(binocular looking time+non-binocular anterior looking time). Here, the non-binocular anterior visual field refers to the anterior visual field, between −90 deg and 90 deg in azimuth, excluding the binocular areas, as shown in Fig. 5D. If we assume random viewing, the chance level can be defined by the size difference between the binocular and non-binocular anterior visual fields, which was determined to be −0.42. To quantify the crows' use of the left and right non-binocular anterior visual fields, we derived a laterality score defined as (right non-binocular anterior looking time–left non-binocular anterior looking time)/(right non-binocular anterior looking time+left non-binocular anterior looking time). Its chance level (in the case of random viewing) was determined to be 0. These scores were calculated as the differential looking score (DLS), which is often used in psychology to analyze bias in looking times (Corkum and Moore, 1998; Kano et al., 2019; Senju et al., 2009).

#### Statistical analyses

To analyze binocular use and laterality scores, we used R (version 4.1.0) and ran a Generalized Linear Mixed Model (GLMM) with a Gaussian error structure and identity link function using the function lmer in lme4 package v. 3.1-3 (https://CRAN.R-project.org/package=lme4).To analyze the binocular use score, the model included the binocular use score as a response variable, the movement of visual targets as a fixed factor, and subjects and sessions as random effects. Because experiment 3 had only one type of object movement (wiggling), we compared the results from experiment 3 with those from wiggling (M1) of experiments 1 and 2 (as between-subject comparisons, with the same GLMM structure). In these analyses, we assessed the significance of the fixed factor by employing a likelihood ratio test, using either the ‘drop1’ or ‘anova’ function in R (https://www.r-project.org/). This involved comparing the full model to a reduced model that excluded the fixed factor.

Additionally, we tested whether the binocular use score and the laterality score deviate from the chance level for each movement within each experiment. For the binocular use score, we first adjusted the chance level (–0.42) to zero by subtracting it from all scores. In the model for both scores, we included the respective score as a response variable, with subjects and sessions as random effects. We then compared the responses to a chance level of zero by testing the significance of the intercept.

In all analyses, we checked the assumptions of normally distributed and homogeneous residuals in the diagnostic plots: histograms of residuals, *Q–Q* plots of residuals, and residuals plotted against fitted values. It is generally recommended that the levels of random factors be greater than five (Gomes, 2022); however, we had only four subjects (and more than six sessions in all experiments). Although this was recently considered acceptable as long as we were interested in testing fixed effects (Gomes, 2022), we also conducted individual-level analyses to confirm the observed effects across subjects. This was done by using the same model without the ‘subject’ as a random factor, or by employing one-sample *t*-tests. For the group-level analyses, we also checked the model stability for the random factor ‘session’ by removing each session independently and calculating Cook's distance each time (‘influence.ME’; ttps://CRAN.R-project.org/package=influence.ME) and found that no session was influential (<1) in all analyses.

## RESULTS

### Visual field configuration

The visual fields of the large-billed crows were reconstructed, as shown in Fig. 1C,D. Note that, in our data, the horizon of the reconstructed visual field derives from their natural perching posture (Fig. S2A), and the beak projects into −10 deg in elevation. When the experimenter did not induce the crows' eye movement, the maximum width of the binocular field (the maximum range in azimuth at any elevation) was 48.4±1.6 deg (mean±s.d. in four subjects) at an elevation of 16.4 deg (no s.d.), and the maximum width of the blind area was 17.1±4.9 deg at an elevation of 176.3 deg (no s.d.). Although we found no s.d. for the elevation at which the maximum width of the binocular field or blind area was observed (the measurements were performed at a step of 1 deg in azimuth and 10 deg in elevation), the calibration of the subjects' heads (the rotation of the subject's head based on its eye-beak-tip line) yielded some variations in the calibrated elevation values (s.d. of ±2.7 deg for both measurements). The maximum amplitudes of the crows' eye movement (with no attempt to induce eye movement) were observed as the range of azimuth values in the measurements of the retinal boundaries; 16.1±8.8 deg for the right eye and 17.3±11.1 deg for the left eye. The mean angle of optical axes was 62.7±1.5 deg in azimuth and −32.9±5.0 deg in elevation for the right eye and 59.4±3.4 deg in azimuth and −33.8±8.7 deg in elevation for the left eye (Fig. 1).

Fig. S1 shows the data from when the experimenter induced eye movements of the crows. When the experimenter induced the crows' eye movement toward the ophthalmoscope, the maximum width of the binocular field was 50.2±4.9 deg at an elevation of 15.6 deg (no s.d.). When the experimenter induced the crows' eye movement away from the ophthalmoscope, we did not observe the binocular field but instead observed a blind area in front of their head; the maximum width of this blind area was 25.1±11.0 deg at an elevation of 75.6 deg (no s.d.). The maximum width of the blind area behind the head was 24.6±3.8 deg at an elevation of 165.5 deg (no s.d.). As noted above, while we report no s.d. for the elevation values, the calibration of the subjects' heads yielded some variations in the calibrated values (s.d. of ±1.75 deg for all measurements). The maximum amplitudes of the crows' eye movement (with attempts of eye movement induction) were 33.9±2.2 deg at an elevation of 15.5±1.8 deg for the right eye and 33.8±0.86 deg at an elevation of 15.6±1.7 deg for the left eye.

### Visual field use

An example of the visual field use of a crow is shown in Fig. 3. In this example, the crow moved its head and used different regions of the visual field to track the distinct movements of the visual targets. When the experimenter took out the visual target from the bag and wiggled it (movement M1), the crow viewed it with its non-binocular anterior visual field. When the object was thrown and followed a parabolic motion (movement M2), the crow continuously tracked it with its binocular field, particularly the region around the beak projection, by smoothly moving its head (Fig. 3). When the object landed on the ground (movement M3), the crow viewed the visual target with its non-binocular anterior visual fields by rotating its head along the roll axis (the *y*-axis in our head-coordinate system) with saccadic head movement.

**Fig. 4.**
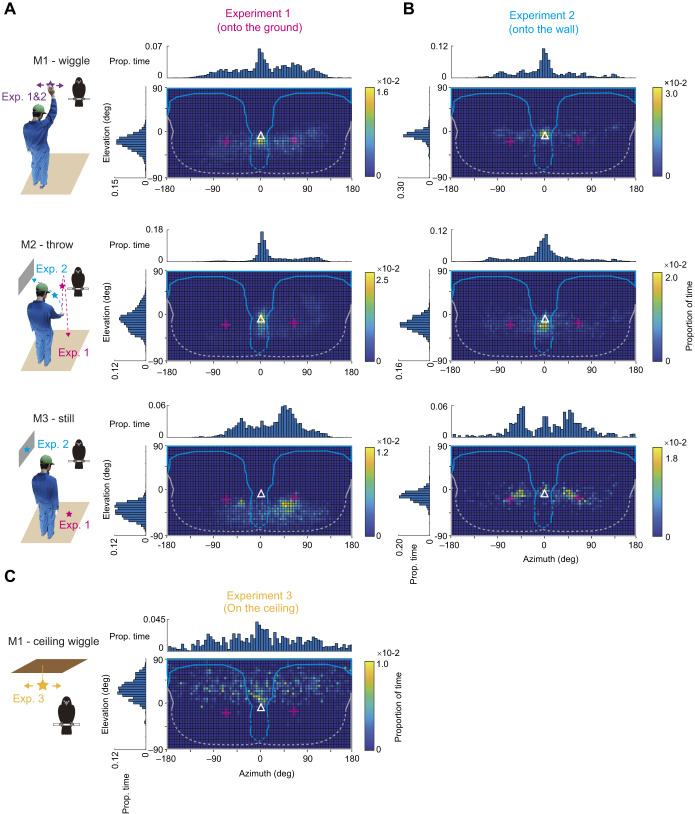
**The distribution of visual targets in the crows' visual fields.** Distribution of visual targets in visual fields in experiments that involved wiggling objects and then throwing them on the ground (experiment 1; A) or onto the wall (experiment 2; B), or wiggling objects on the ceiling (experiment 3; C). The three rows in A and B show the distinct movements (M1–M3) of the visual target, with an illustration on the left. Each bin was 5×5 deg and indicates the proportion of time when visual targets were observed in this bin. In addition, histograms (binned at 5 deg) plot the same data in azimuth and elevation. The reconstructed visual field from study 1 (Fig. 1E) was superimposed onto each heat map. The plus marks denote the optical axes in study 1. Blank triangles denote the projection of the beak-tip (eye–beak-tip line).

To visualize the visual field use of all crows, we mapped the distribution of visual targets in their head-centric coordinate system as a heatmap (with the pooled data across individuals) for each experiment (experiment 1–3) and target movement (M1–M3) and mapped the reconstructed visual field from study 1 (Fig. 1E) onto this heatmap (Fig. 4). The heatmaps suggested that the crows mainly oriented their anterior visual field (between −90 deg and 90 deg in azimuth) and oriented distinct regions of the anterior visual field to the visual target depending on the target's movements when wiggled and thrown on the ground or wall (experiments 1 and 2). Specifically, they tended to orient the binocular rather than non-binocular field to the visual target during wiggling (M1), oriented the binocular field to the visual target even more frequently during throwing (M2) and then mainly oriented the non-binocular anterior visual field to the visual target when it was still (M3). When they oriented the binocular field to the visual target, they tended to orient the regions around the beak projection (the eye–beak-tip line); and when they oriented the non-binocular anterior visual field to the visual target, they tended to orient the regions around where we found the optical axes. When an object was wiggled on the ceiling (experiment 3), the crows tended to orient the binocular field to the visual target. However, in this case, crows showed a weaker tendency to use a specific visual field compared with the distribution during M1 before throwing target to the ground or the wall.

**Fig. 5.**
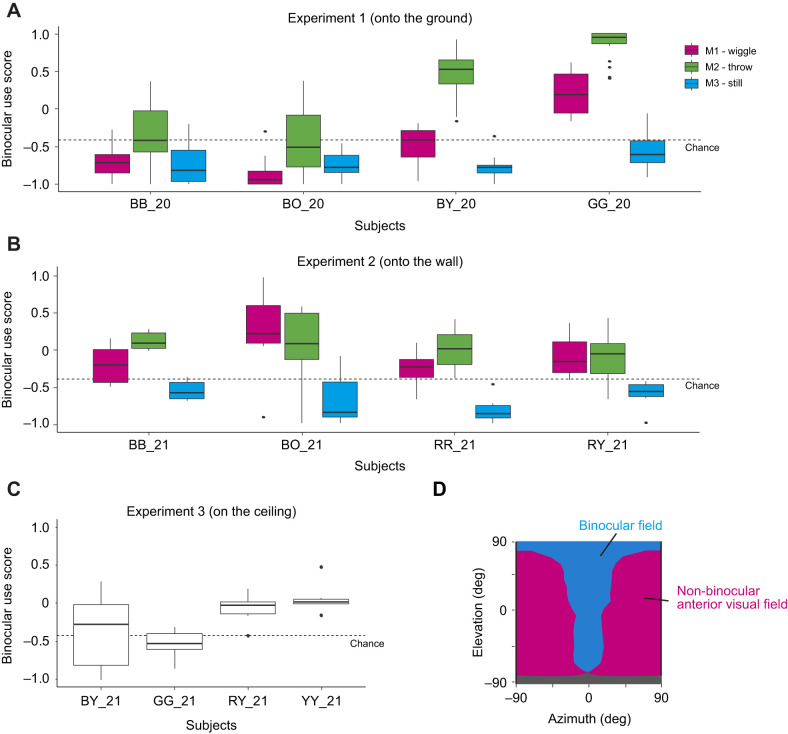
**Binocular use scores in each experiment.** Binocular use scores were defined as (binocular looking time×non-binocular anterior looking time)/(binocular looking time+non-binocular anterior looking time) in experiment 1 (A), experiment 2 (B) and experiment 3 (C). Box plots show the median, interquartile range (IQR) and 1.5×IQR, with outliers plotted individually. (D) In addition, the 2D visual field map created in study 1 was used to calculate the binocular use score; the binocular field, non-binocular anterior visual field and blind area are indicated in cyan, magenta and gray, respectively. The chance level was calculated based on the size of binocular field and non-binocular anterior visual field and set to −0.42.

To examine the crows' differential use of binocular fields across the targets' movement and experiments, we calculated the binocular use score by comparing the time spent looking with the binocular field and the non-binocular anterior visual field (Fig. 5). The binocular use score varied depending on the movement of the visual target onto the ground and onto the wall (likelihood ratio test, 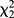
=8.1, *P=*0.017 for experiment 1; 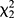
=13.3, *P=*0.001 for experiment 2). It was the largest for throwing (M2), followed by wiggling (M1) and then while still (M3) (likelihood ratio test, 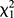
=2.1, *P=*0.148 for M1 and M2; 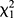
=13.1, *P=*0.001 for M2 and M3; 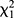
=3.9, *P=*0.049 for M3 and M1). We performed the same analysis (the same GLMM without the subjects as a random effect) at the individual level and found that all crows showed similar results (the largest *P*-value was 0.007).

When random viewing was assumed as a chance level, crows demonstrated a clear preference for the non-binocular anterior visual field when the object was still on both the ground and the wall (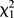
=10.2, *P=*0.001 for experiment 1; 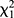
=8.5, *P=*0.004 for experiment 2). Conversely, they exhibited a preference for the binocular field when the object was wiggled or thrown onto the wall (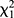
=4.3, *P=*0.039 for M1; 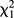
=9.4, *P=*0.002 for M2). However, this tendency was not clear when the object was wiggled or thrown onto the ground (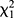
=0.1, *P=*0.781 for M1; 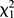
=2.9, *P=*0.091 for M2) due to the individual variations in the extent of this preference (Fig. 5).

As experiment 3 contained only one movement of the target on the ceiling, we compared the binocular use score here with that of a similar condition, wiggling of the target (M1) before throwing to the wall or ground, using a between-subject analysis (with the same GLMM structure). No significant differences between the experiments were found in these analyses (likelihood ratio test, 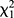
=1.0, *P=*0.312 for experiments 1 and 3 and 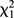
=0.3, *P=*0.585 for experiments 2 and 3). All birds exhibited similar responses across trials, indicating that habituation was minimal (Fig. S4).

**Fig. 6.**
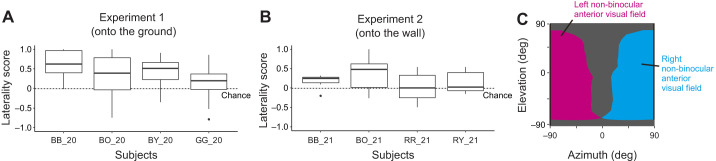
**Laterality scores in each experiment.** Laterality scores were defined as (right non-binocular anterior looking time-left non-binocular anterior looking time)/(right non-binocular anterior looking time+left non-binocular anterior looking time) in experiment 1 (A) and experiment 2 (B). Box plots show the median, interquartile range (IQR) and 1.5×IQR, with outliers plotted individually. Only the sessions after visual targets landed on the ground or wall were tested because crows mainly use the non-binocular anterior visual field when objects were still. The dotted line indicates the chance level (zero). The 2D visual field map created in study 1 was used to calculate the laterality score (C). The right and left sides of the non-binocular anterior visual field are indicated in cyan and magenta, respectively.

To examine the laterality preference in eye use, we calculated the laterality score by comparing the time spent looking with the right non-binocular anterior visual field and the left non-binocular anterior visual field when targets were still on the ground and the wall (Fig. 6). The crows showed a right-eye preference for targets on the ground (likelihood ratio test, 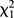
=7.0, *P=*0.008) and a similar trend for targets on the wall (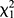
=4.2, *P=*0.040). We also tested the laterality bias within each individual and found that three out of the four crows showed significant right-eye preferences for targets still on the ground (the *P*-value for GG_20 was 0.159, whereas the largest *P*-value was 0.009 for the other crows) but no crows showed a significant preference when targets were still on the wall.

## DISCUSSION

This study examined the visual field use of large-billed crows. Our main objectives were twofold: first, to identify the visual field regions they orient to when presented with an attention-getting visual target, and second, to explore the contextual cues influencing the differential use of their left and right eyes, as well as distinct visual field regions. By addressing these objectives, we aimed to determine the feasibility of inferring a crow's attentional focus from its head orientation.

With the visual field configuration data measured in study 1, we found that when crows were presented with a distal visual target, they used limited regions of the visual field, particularly the region around the beak projection (eye–beak-tip line), which is within the binocular region, as well as the regions around where we found the optical axes in study 1, which is within the non-binocular region. The latter regions are likely where the area centralis, the most visually sensitive spots in their retina (Rahman et al., 2006), projects, close to their visual axes. In our experiments, the selective use of the binocular field was mainly affected by the movement properties of the object in large-billed crows. Specifically, when the experimenter wiggled the object, crows tended to orient their binocular field to the object. When the experimenter threw the object, crows increased this tendency and typically followed the object's movement via the smooth movement of their head (see Fig. 3). When the object landed on the ground or wall, crows tended to orient their non-binocular visual fields (likely their visual axes) to the object, typically via the saccadic movement of the head. They preferred to use the right eye when orienting their non-binocular visual fields to the object. When the objects suspended on the ceiling were wiggled, crows showed weaker tendency to use a specific visual field than when objects were wiggled by the human experimenter, presumably because the visual targets on the ceiling attracted the crows' attention less strongly.

### Visual field configuration

How does the visual field configuration of this species compare with that of other corvid species? The maximum binocular overlap in this species was approximately 50 deg, regardless of whether the experimenter attempted to induce eye movements. This observation is largely comparable to those of other corvid species, which vary between 37.6 deg and 61.5 deg (Fernández-Juricic et al., 2010; Troscianko et al., 2012). The maximum eye movement amplitude was approximately 16–17 deg when we did not induce eye movement and 33 deg when eye movement was induced. The measurement of 16–17 deg (eyes at rest) is smaller than that of any other corvid species reported by Troscianko et al. (2012), who did not induce eye movement in corvids, but the measurement of 33 deg is largely comparable to that of other corvid species (22.2–38.8 deg). Owing to large eye movements, the binocular field was temporarily lost when the eyes were attracted away from the ophthalmoscope, and a blind area was observed in the same visual field region. This observation is largely consistent with previous studies that tested several other passerine species, such as the American crow (Fernández-Juricic et al., 2010), American goldfinch (Baumhardt et al., 2014), chickadee (*Poecile carolinensis*) and titmouse (*Baeolophus bicolor*) (Moore et al., 2013).

### Visual field use

Why did crows prefer to use their binocular field when attending to an object in motion? Their binocular use is unlikely to be related to the use of area centralis because their optical axes (located at the azimuth of approx. 60 deg) cannot project into the binocular fields (50 deg in width, i.e. ±25 deg from the front), even when considering the largest eye movements we observed (30 deg).

Rahman et al. (2006) reported that this species has a relatively high density of retinal ganglion cells in the dorso-temporal part of the retina, which projects forward. Thus, the binocular use of crows may be due to the availability of relatively high-resolution visual information as a result of the high density of ganglion cells. An alternative possibility is that they use optical flow field information from the binocular field, such as the moving direction and time to contact a visual target. In birds, it is generally assumed that such information is obtained more efficiently from the binocular field than from the monocular field because the optical flow field appears symmetrical when a target object is located in the binocular field (Martin, 2009). Moreover, motion perception may be more fine-tuned in the binocular field because it may contain a relatively higher ratio of double to single cones in the retina, as found in several bird species (Fernández-Juricic et al., 2019; Tanaka, 2015).

### Right eye bias

Our crows were slightly biased toward using their right eye when viewing the presented visual target. Right eye bias (left hemispheric bias) is generally observed in birds when they attend to the detailed features of objects (Clayton and Krebs, 1994; Rogers, 2008). As our visual targets were small objects of various shapes and colors, our crows were likely motivated to attend to the detailed features of the objects. It should be noted that, in our experiments, the observed eye preference was weaker in when objects were thrown on the wall compared with the floor. One reason for this could be that the object tended to land on the ground in front of the crows, whereas it could land on any one of four sides of the wall (at approximately the eye level of the crows), so it may have been physically easier for crows to select the preferred eye when the object was on the ground. Thus, although our crows generally prefer to use their right eye, such a preference could easily be lost when there are certain physical constraints. In addition, although three out of the four crows showed a significant right eye bias for objects on the ground, it is important to note that previous research, including studies involving this species, has reported individual differences in the laterality bias among corvids (Clary et al., 2014; Izawa et al., 2005; Mack and Uomini, 2022). Therefore, caution is warranted when generalizing our results across individuals.

### Eye movement

We can assume that our motion capture system accurately tracked crows' head orientations (Itahara and Kano, 2022). Thus, eye movements may largely explain variations in the distribution of objects in the visual field. Visual inspection of the heatmaps in study 2, particularly Fig. 4A,B, suggests that eyes regularly shift the optical axes by approximately 15 deg, which is consistent with our observations in study 1, where we observed 16–17 deg of eye movement when not attempting to induce eye movement (33 deg when inducing eye movement, possibly from one extreme angle to another). Fig. 4A,B indicates that the optical axis tends to shift both upward and downward in elevation but mostly inward in azimuth (the direction toward the beak). This suggests that crows may have mainly converged, rather than diverged, their eyes in our experiments. Typically, birds' eyes converge when viewing a close object within their binocular field (Bloch et al., 1984; Troscianko et al., 2012).

**Fig. 7.**
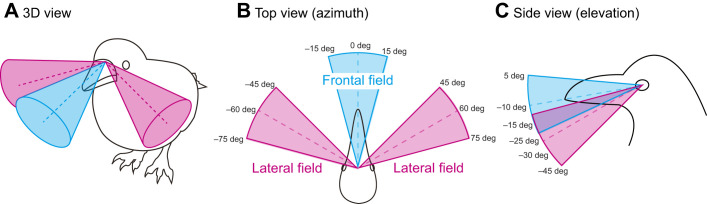
**The simplified visual field model of large-billed crows.** The frontal field around the projection of the beak-tip (eye–beak-tip line, cyan) and the lateral field around the optical axes (magenta) were drawn from oblique (A), top-down (B) and side views (C). Dotted lines indicate the angle of the beak-tip projection (in cyan) and optical axes (in magenta).

Our additional analysis, shown in Fig. S5, examined the conditions under which crows might have used their convergent eyes to view a distant object with non-binocular visual fields. This analysis suggested that the peak in the heatmap was approximately 15 deg more inward when crows first followed the object with their binocular field and then oriented their visual axes (close to their optical axes) to the objects, compared with when crows viewed the object directly with their visual axes. This suggests that the crows first converged their eyes to view the object with the binocular field and then maintained these eye positions to fixate on the object near the optical axes. Overall, it appears that crows commonly exhibited eye movements within 15 deg in our experiments. Taking into account the visual field configuration and untracked eye movements, a simplified model of a large-billed crow's visual field can be depicted as shown in Fig. 7.

### Limitation of our tracking method

One limitation of our tracking method is that the motion capture system requires the attachment of a few lightweight reflective markers to the subject's body. Although most of our subjects accepted the marker attachments, we could not test several subjects because they habitually removed the attached markers on their heads with their claws (see the Materials and Methods). The use of a head-mounted camera (Kane et al., 2015; Kane and Zamani, 2014) or eye tracker (Schwarz et al., 2013; Yorzinski et al., 2015; Yorzinski and Platt, 2014) could significantly improve gaze-tracking accuracy. However, it may not be practical with corvid species, as they can easily remove such devices. Recent developments in marker-less tracking methods (e.g. Dunn et al., 2021; Nath et al., 2019; Pereira et al., 2022; Waldmann et al., 2022) offer potential solutions to the attachment issue. This marker-less method should also help in reducing the bird's stress associated with the restraint required for marker attachment. Nevertheless, it is essential to carefully consider the trade-off between accuracy and applicability when choosing between infrared motion capture systems, which are generally more accurate, and marker-less tracking methods.

### Conclusion

Overall, our results indicate that crows exhibit predictable patterns for their visual field use, thus demonstrating the feasibility of inferring their attentional focus through 3D tracking of their head. Owing to untracked eye movements, there is an estimated margin of error of 15 deg around their visual axis (Fig. 7). The differential use of their binocular and non-binocular fields likely depends on the motion of an object, according to our study, and probably also on the distance between the bird and an object, according to a previous study (Matsui and Izawa, 2019). The differential use of their left and right, although a weak tendency, may partly depend on the type of objects. Further investigation is required to explore what other contextual cues influence the differential use of their binocular or non-binocular fields and left and right eyes.

While more work is necessary, our current system represents a promising initial step towards establishing gaze-tracking methods in the studies of corvid behavior and cognition. Notably, our specific procedures to test the possibility of gaze tracking can be applicable to other corvid and non-corvid bird species as well. Moreover, although this study tracked one bird at a time, our system can simultaneously track multiple individuals. As a result, it can be utilized to investigate socio-cognitive behaviors in birds, such as eye contact, gaze following and theory of mind, within an interactive setup.

## Supplementary Material

10.1242/jexbio.246514_sup1 Supplementary information

## ECR Spotlight

This article has an associated ECR Spotlight interview with Akihiro Itahara.
