# Supplementary material for: Gaze tracking of large-billed crows (Corvus macrorhynchos) in a motion capture system
Source: J Exp Biol. 2024 Mar 22;227(6):jeb246514. doi: 10.1242/jeb.246514 (PMC11007591; doi:10.1242/jeb.246514)
Supplement: Supplementary information [file jexbio-227-246514-s1.pdf]

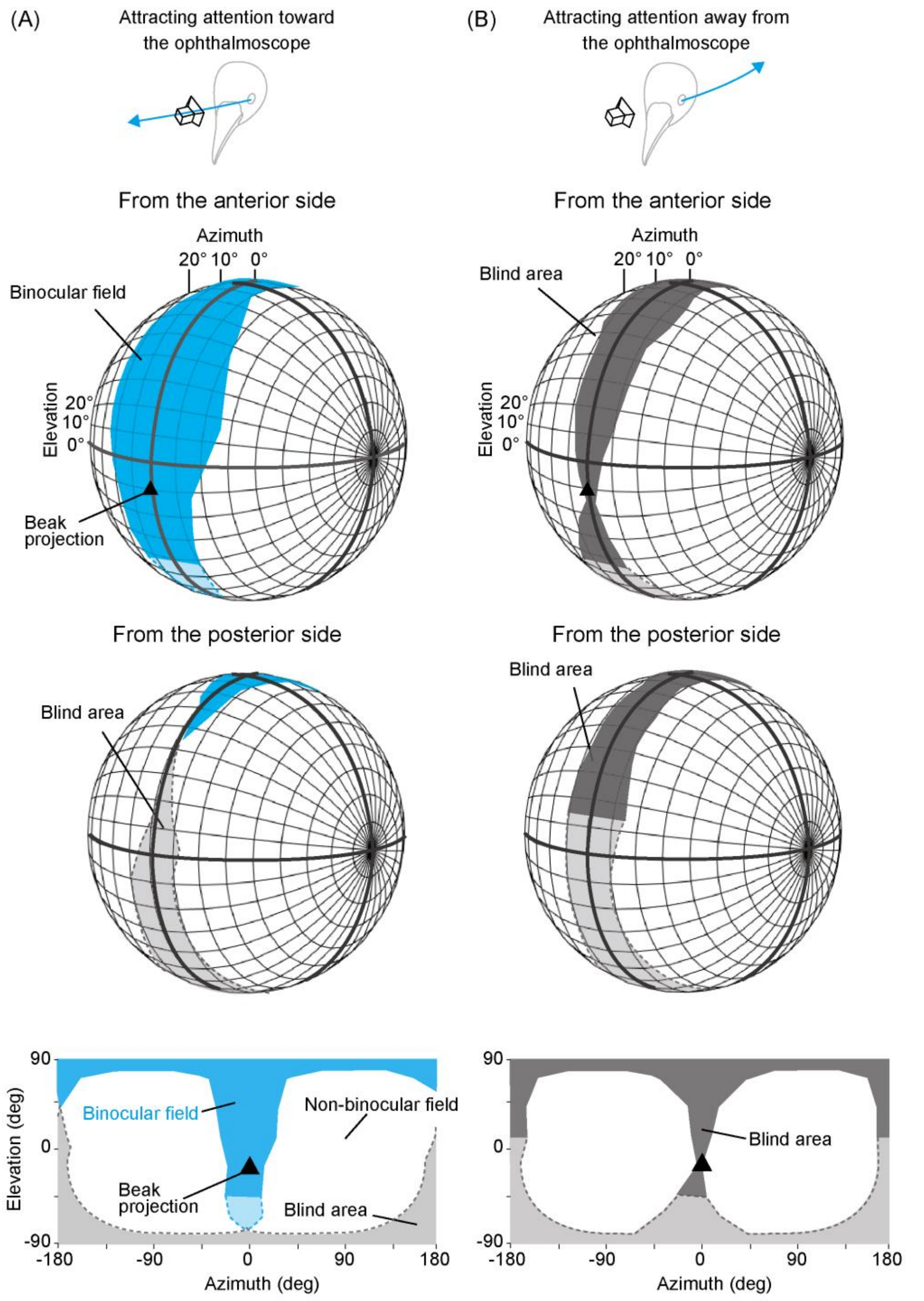

Fig. S1. Visual field configurations of large-billed crows when eye movement was induced.

3D illustrations of the visual field in Study 1 when the experimenter attracted the subject's attention toward (A) and away from the ophthalmoscope (B) along the perimeter arm (azimuth axis). The projection of the beak-tip (eye-beak-tip line) is denoted by a filled triangle. Manual interpolations are indicated by dotted lines and lighter colors. 3D illustrations of the visual field configuration were mapped in 2D at the bottom panels.

Journal of Experimental Biology • Supplementary information

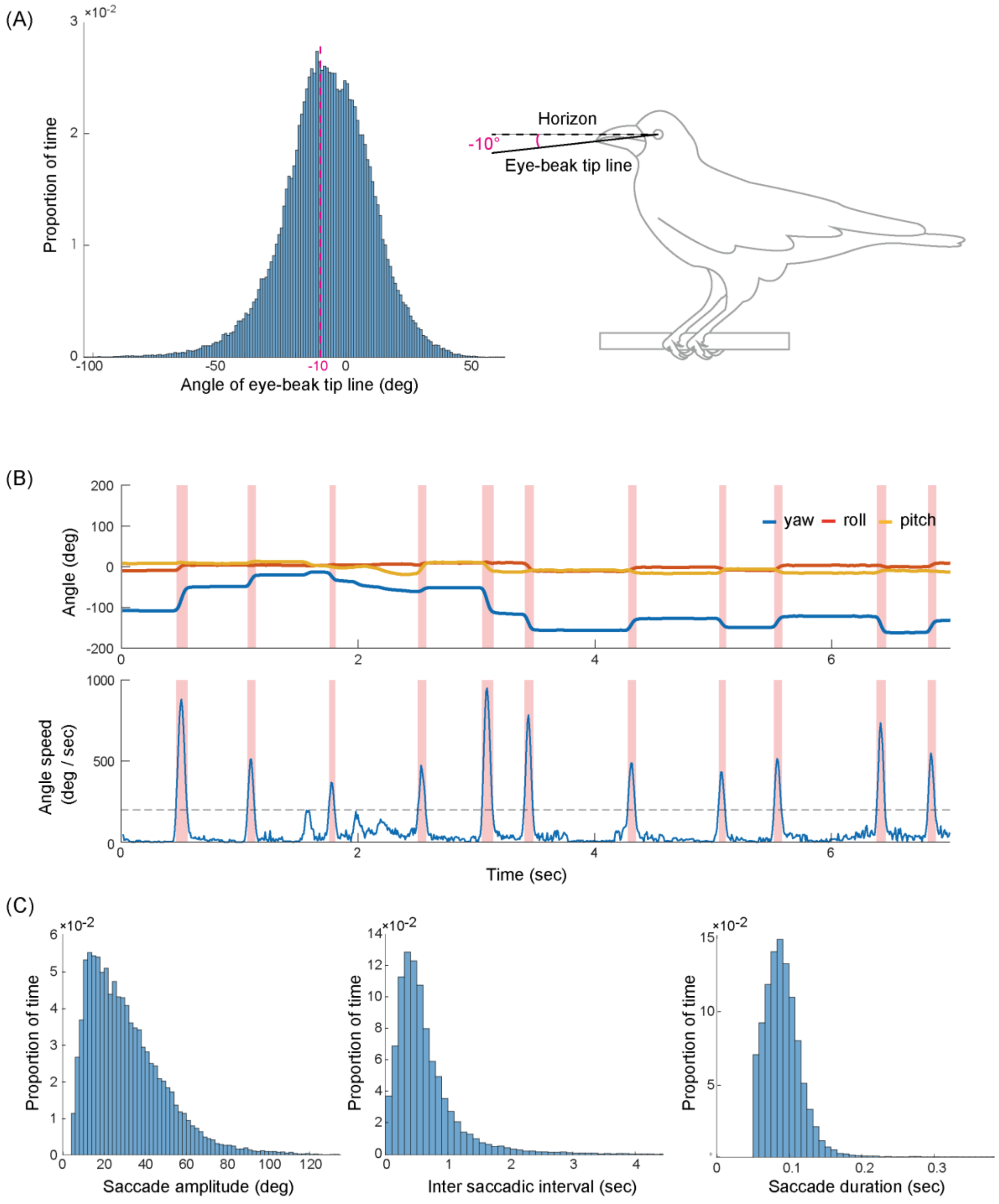

Fig. S2. The metrics of the head angle and movement in large-billed crow.

(A) The proportion of the elevation angle (°) of the eye-beak tip line from the horizontal line (per 1° bin). (B) An example of rotational movement of the crow's head in yaw, roll, and pitch (top) and axial angle speed (bottom). Saccades are highlighted in magenta. (C) Histograms of saccade amplitude (°), inter saccadic interval (sec), and saccade duration (sec).

Journal of Experimental Biology • Supplementary information

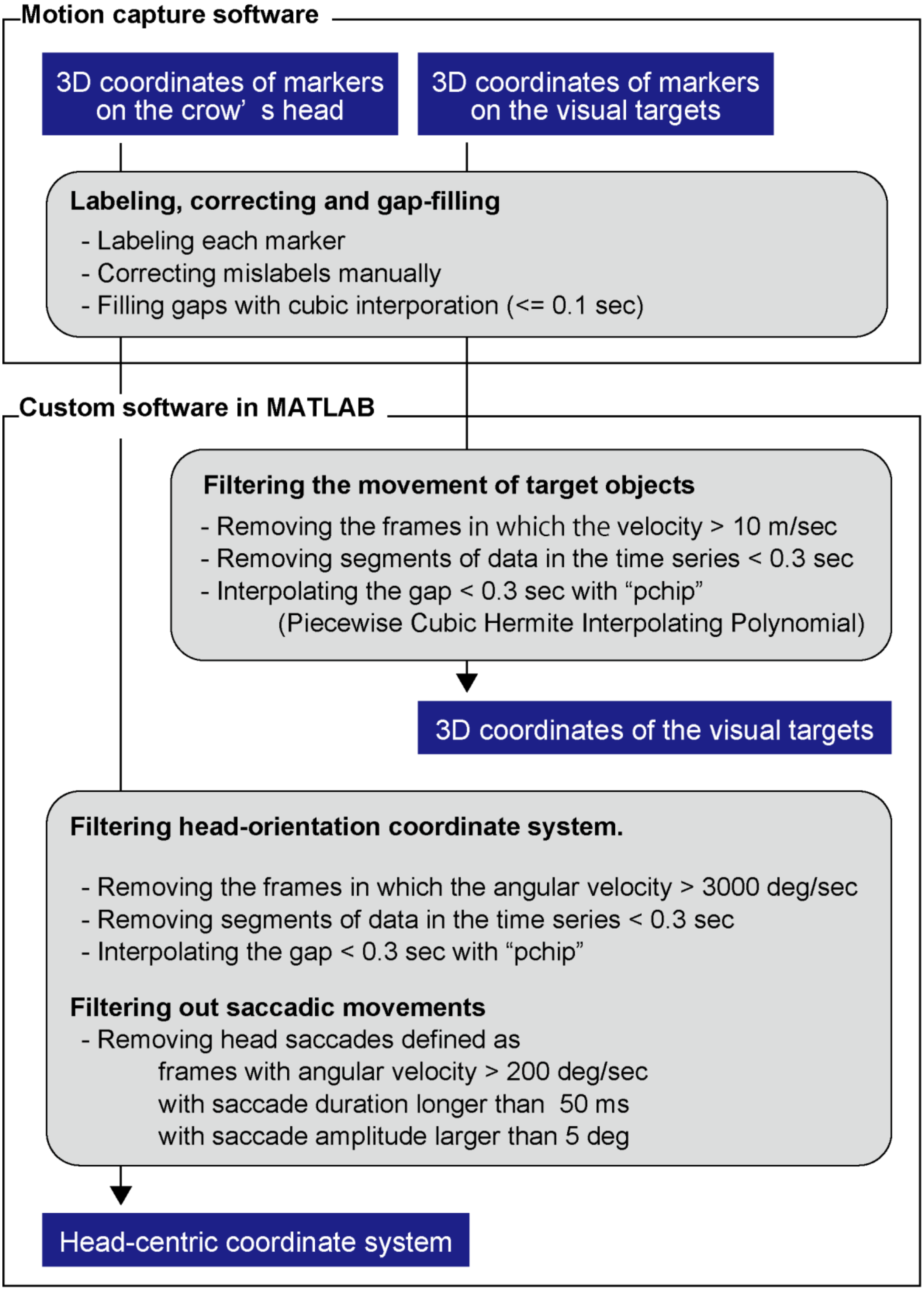

Fig. S3. The data-filtering pipeline.

Journal of Experimental Biology • Supplementary information

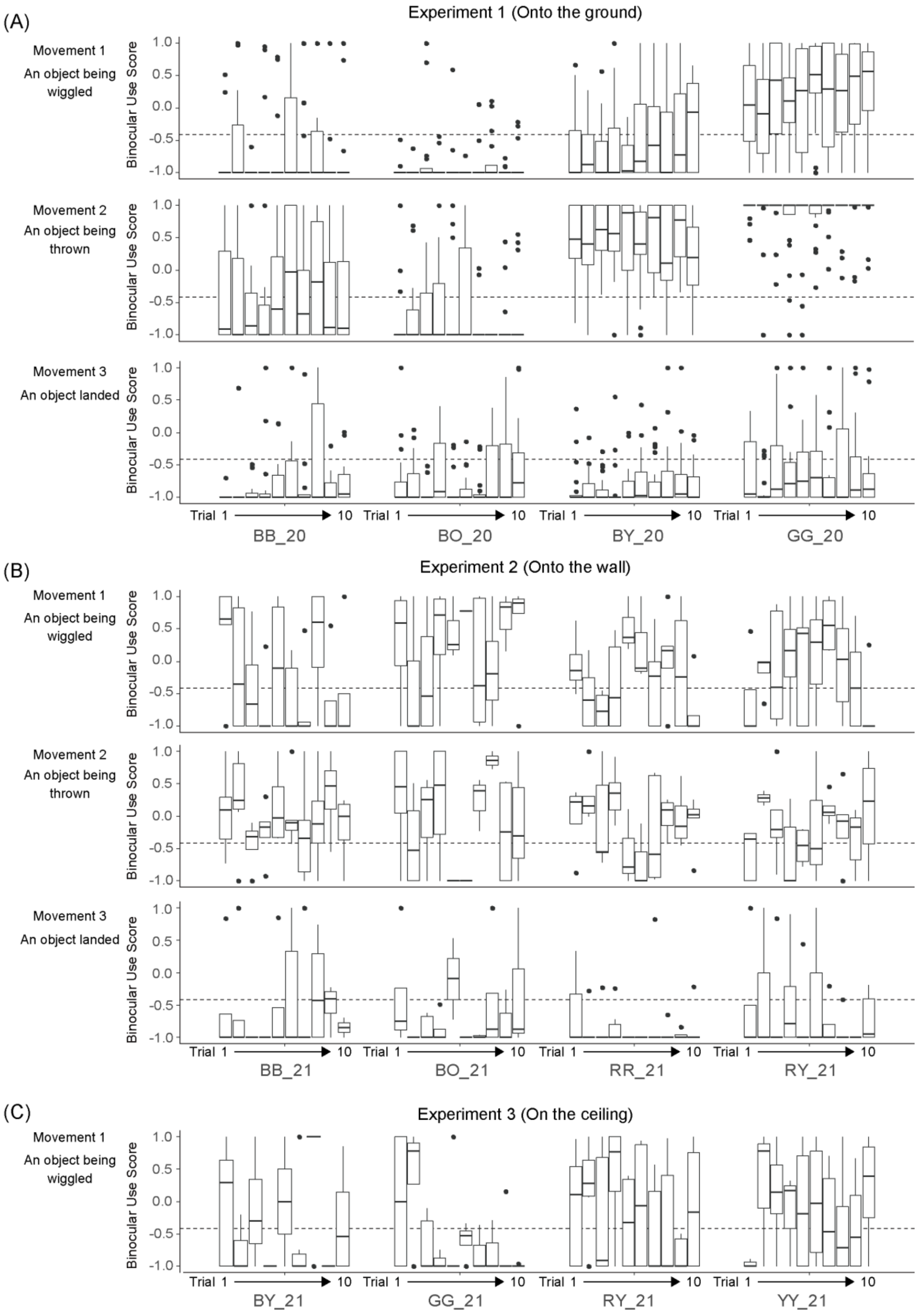

Fig. S4. Binocular use scores arranged by the order of trials.

To assess habituation due to successive object presentations, the binocular use scores were arranged by the order of trials in Experiments 1 (A), 2 (B), and 3 (C). The 10 trials per session for each individual were arranged from left to right. Box plots show the median, interquartile range (IQR), and 1.5 × IQR, with outliers plotted individually.

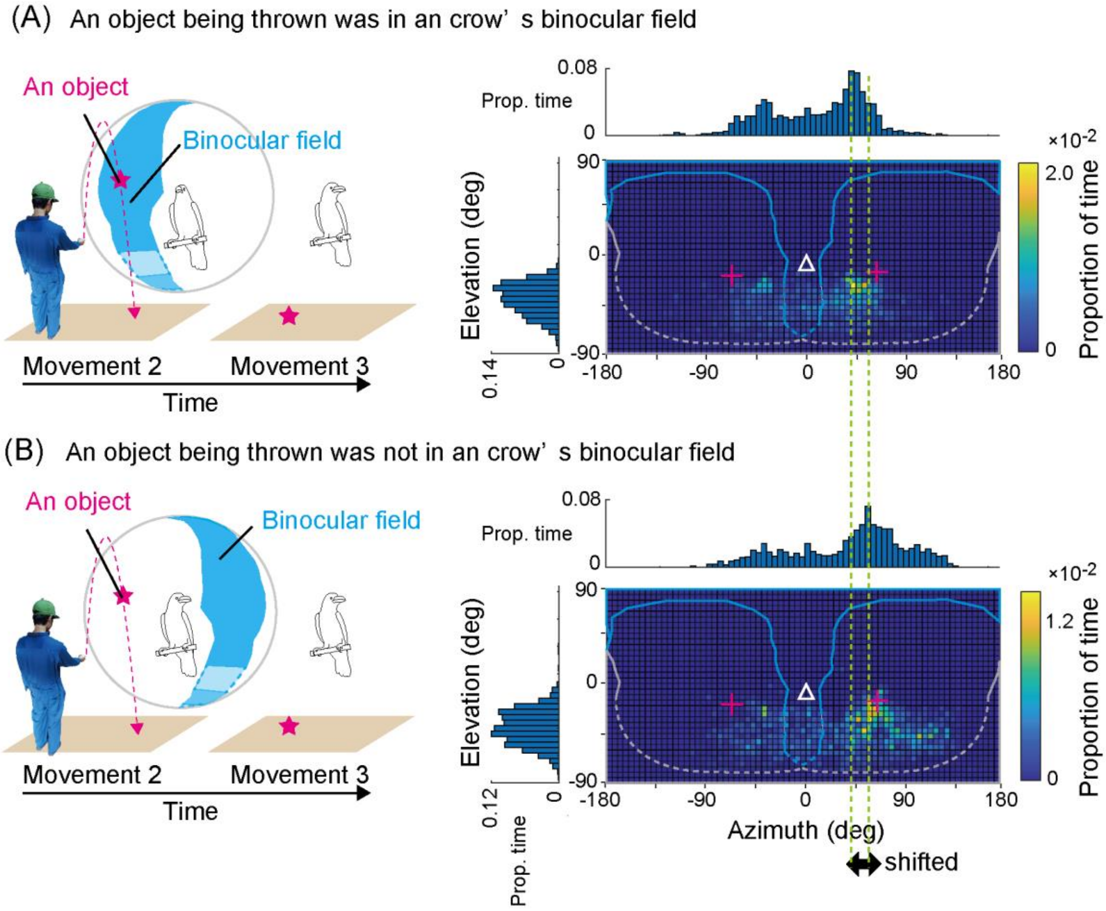

Fig. S5. Analysis of one potential situation where crows used their eyes differently when viewing the presented visual target.

Heatmaps were drawn in the same manner, as shown in Figure 4. Specifically, we compared the difference in the peak distributions of visual targets around the optic axes of the crows between the cases in which they first oriented their binocular field when the object was thrown by the experimenter (in more than half of the frames during the 1-sec time window) and then oriented their non-binocular anterior visual field when the object landed on the ground (A) and the case in which they oriented their non-binocular anterior visual field throughout (B). The heat maps in A and B (the distribution of visual targets, binned 5° × 5°) show the distribution of visual targets when the visual target landed on the ground (the crows tended to view the targets with their non-binocular anterior visual field, especially around the optic axes). We reasoned that the difference in the peaks of these distributions around the optic axes potentially indicates the amplitude of eye movement; more specifically, this is because, in the former case, the crows likely used their semi-converged eyes to view the visual target, and, in the latter case, they likely used their diverged eyes throughout. We observed that the potential difference in eye orientation was approximately 15°.

Journal of Experimental Biology • Supplementary information

Table S1. Details about the participants.

Participant	Sex	Age*	Year captured	Captured at	Participated in
BB_20	M	2-3	2018	The study station	Study 2 (Exp. 1)
BO_20	F	2-3	2018	The study station	Study 2 (Exp. 1)
BY_20	F	2-3	2018	The study station	Study 2 (Exp. 1)
GG_20	F	2-3	2018	The study station	Study 2 (Exp. 1)
BB_21	M	1-2	2021	Gyokuto-town	Study 2 (Exp. 2)
BO_21	M	1-2	2021	Gyokuto-town	Study 1, Study 2 (Exp. 2)
BR_21	F	1-2	2021	The study station	Study 1
BY_21	M	1-2	2021	Gyokuto-town	Study 1, Study 2 (Exp. 3)
GG_21	F	1-2	2021	Gyokuto-town	Study 2 (Exp. 3)
RR_21	F	1-2	2021	Gyokuto-town	Study 2 (Exp. 2)
RY_21	M	1-2	2021	Gyokuto-town	Study 2 (Exp. 2 & Exp. 3)
YY_21	M	1-2	2021	Gyokuto-town	Study 1, Study 2 (Exp. 3)

* Age at the time of study

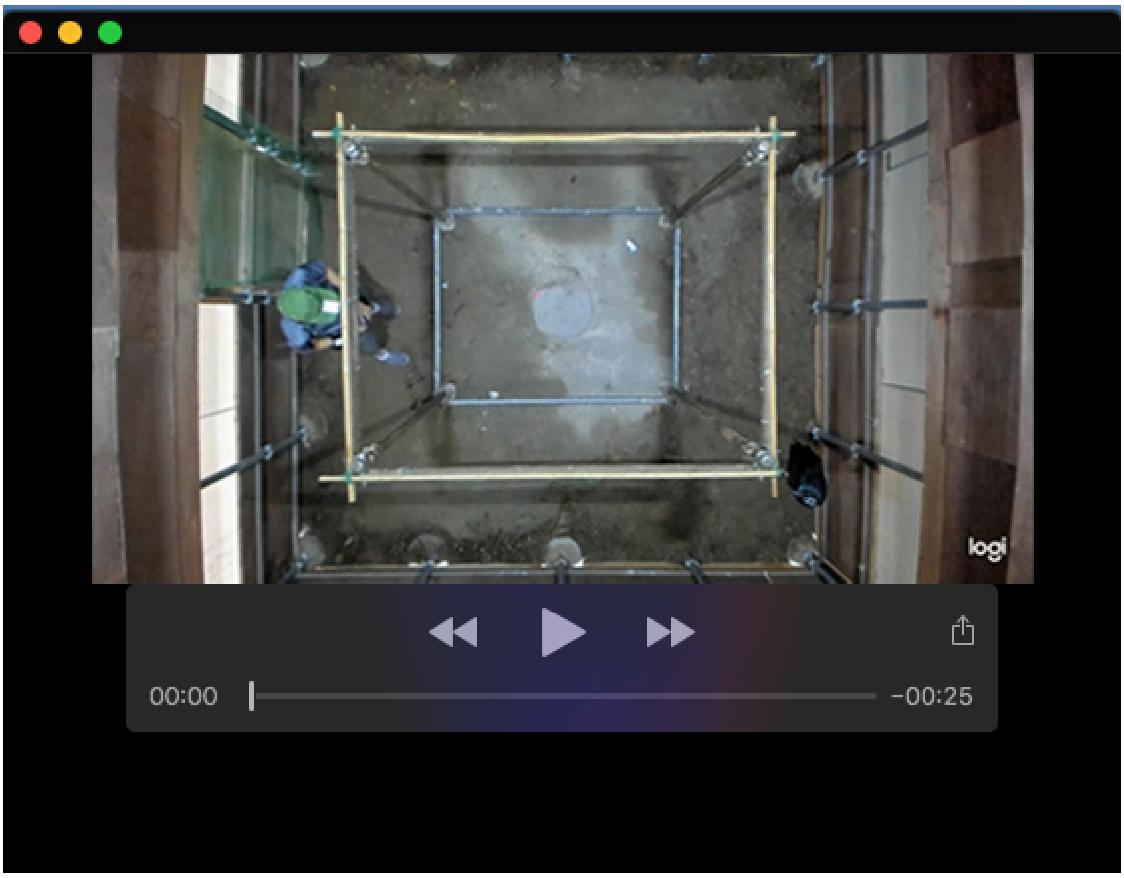

Movie 1. The sample movie for Experiment 1 and Experiment 2.
